# Single and combinatorial chromatin coupling events underlies the function of transcript factor krüppel-like factor 11 in the regulation of gene networks

**DOI:** 10.1186/1471-2199-15-10

**Published:** 2014-05-25

**Authors:** Ezequiel Calvo, Adrienne Grzenda, Gwen Lomberk, Angela Mathison, Juan Iovanna, Raul Urrutia

**Affiliations:** 1 Molecular Endocrinology and Oncology Research Center, CHUL Research Center, Quebec, Canada; 2 Laboratory of Epigenetics and Chromatin Dynamics, Mayo Clinic, Rochester, MN 55905, USA; 3 INSERM U.624, Stress Cellulaire, 163 Avenue de Luminy, Case 915, Parc Scientifique et Technologique de Luminy, 13288 Marseille, Cedex 9, France; 4 Translational Epigenomics Program, Center for Individualized Medicine (CIM), Mayo Clinic, Rochester, MN 55905, USA; 5 Departments of Medicine, Physiology and Biochemistry, Mayo Clinic, 200 First Street SW, Guggenheim 10, Rochester, MN 55905, USA

**Keywords:** Krüppel-like factor, Transcription factor, Gene expression profiling, Gene networks, Metabolism, Cellular growth, Proliferation, Signaling pathways

## Abstract

**Background:**

Krüppel-like factors (KLFs) are a group of master regulators of gene expression conserved from flies to human. However, scant information is available on either the mechanisms or functional impact of the coupling of KLF proteins to chromatin remodeling machines, a deterministic step in transcriptional regulation.

**Results and discussion:**

In the current study, we use genome-wide analyses of chromatin immunoprecipitation (ChIP-on-Chip) and Affymetrix-based expression profiling to gain insight into how KLF11, a human transcription factor involved in tumor suppression and metabolic diseases, works by coupling to three co-factor groups: the Sin3-histone deacetylase system, WD40-domain containing proteins, and the HP1-histone methyltransferase system. Our results reveal that KLF11 regulates distinct gene networks involved in metabolism and growth by using single or combinatorial coupling events.

**Conclusion:**

This study, the first of its type for any KLF protein, reveals that interactions with multiple chromatin systems are required for the full gene regulatory function of these proteins.

## Background

The traditional strategy for studying the role of transcription factors in gene regulation relies on either small or large-scale expression analysis following overexpression, somatic knockdown, or germ line deletion. While these approaches permit the identification of gene networks regulated by transcription factors, little information is gathered about the chromatin coupling events that ultimately drive gene expression. This gap in knowledge is unfortunate as transcription factors are often modular proteins armed with multiple sites for potential interactions with chromatin cofactors. Thus, knocking-out a transcription factor will, in theory, disrupt all chromatin-coupling events. For this reason, it is important to dissect whether different transcriptional regulators use single or combinatorial coupling events to regulate distinct gene expression networks.

This knowledge is particularly important for proteins in which dysfunction contributes to disease. Impairment in each of the coupling events may yield differential effects, affecting disease penetrance as well as progression. Moreover, with the development of novel chromatin-centric pharmacology, different drugs can partially inactivate certain gene networks regulated by a transcription factor while leaving others intact. Therefore, the dissection of individual and combinatorial chromatin coupling events possesses both biological and medical relevance.

Consequently, the current study focuses on KLF11 as a model system for addressing these important questions. KLF11 is a well characterized human disease-causing gene that couples to several chromatin partners [1]. Alterations in KLF11, originally discovered by its role in growth regulation, causes juvenile (MODY7) and neonatal (Ins-331 mutation) diabetes [2-4]. KLF11 is an inducible gene, responsive to a large variety of growth regulatory and metabolic stimuli, and functions in the nucleus to regulate gene expression by coupling to distinct chromatin partners. Upon stimuli, KLF11 binds to promoters containing the consensus CCCCGC/CCCCAC sequences via its three C-terminal zinc finger domains and, through well-characterized protein-protein interaction modules in its N-terminus, differentially recruits chromatin partners such as the Sin3-HDAC complex [5], WD40 proteins [6], and the HP1-HMT system [7]. Thus, through these domains, KLF11 translates environmental signals into distinct programs of gene expression, which remain to be defined in detail.

In the current study, we have employed a combination of genome-wide ChIP-on-Chip and gene expression profiles to reconstruct both the direct and indirect effects of KLF11 on the regulation of different gene networks. Furthermore, we used site-directed mutants to disrupt individual chromatin coupling domains to generate genome-wide expression for the identification of the genes regulated by KLF11 in response to binding to each of its chromatin cofactors. Our results demonstrate that certain gene programs require a single chromatin machine interaction with KLF11 while others require interaction with multiple chromatin systems. Accordingly, we provide analyses of the distinct gene networks regulated by KLF11 and its coupling to chromatin partners. This type of comprehensive genome-wide analysis has never been performed for any member of the KLF family. When applied to KLF11, our analysis demonstrates that this protein behaves in a modular fashion with chromatin machinery to alter gene expression and impacts a variety of biological processes.

## Results

### Distinct gene expression networks are regulated by the differential coupling of KLF11 to individual chromatin partners

**Figure 1:**
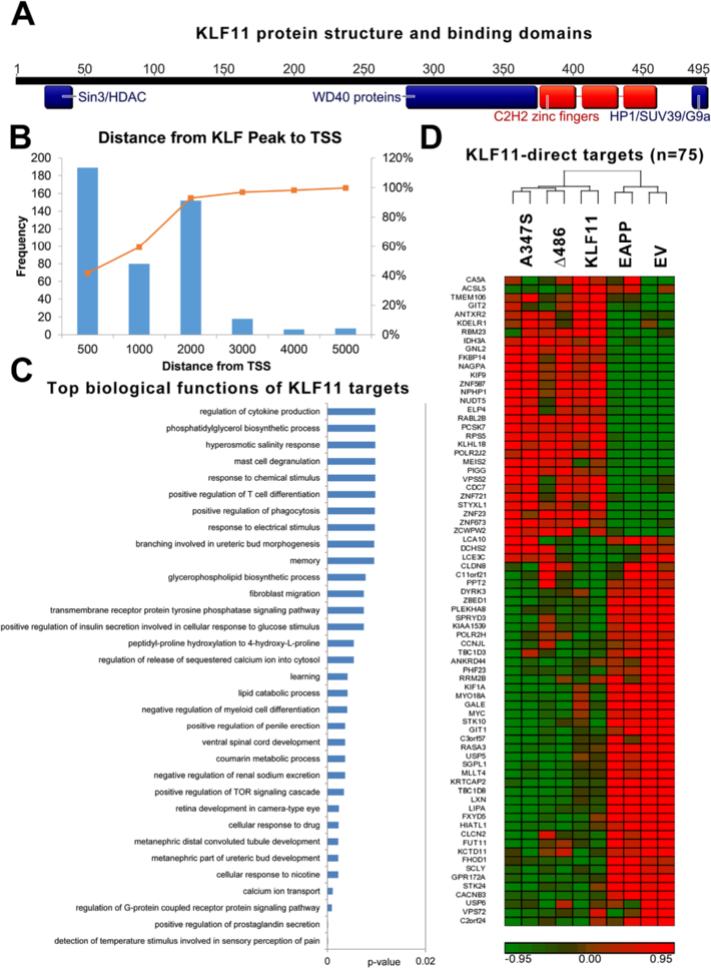
**KLF11-mediated gene expression is disrupted in the presence of chromatin coupling mutations. (A)** KLF11 is 495 amino acids and contains a highly conserved C-terminal C_2_H_2_ zinc finger domain. Known chromatin coupling domains explored here are highlighted. **(B)** Panc1 epithelial cells were transfected with wild type KLF11 and chromatin immunoprecipitation performed and hybridized to a whole genome promoter array. The frequency of identified peaks 5 kb upstream and downstream of known transcription start sites were binned according to distance. The majority of KLF11 binding sites were 500–1000 base pairs from transcription start sites. **(C)** Gene ontological analysis of KLF11-bound targets reveals enrichment of genes in known KLF11-associated biological processes, including immune response, TOR signaling, and insulin sensitivity. For whole genome analysis, mutants were designed against three of the previously characterized chromatin coupling domains. The EAPP mutation in the N-terminus decouples the Sin3/histone deacetylase system. The A347S mutation in the proline rich domain decouples KLF11 from WD40 containing proteins. Finally, the deletion mutation starting at amino acid 486 disconnects KLF11 from the HP1/histone methyltransferase system. Panc1 cells were transduced with empty vector, wild type KLF11 or the A347S, Δ486, or EAPP mutants. Whole genome transcriptional profiling was performed. The criteria for significant regulation over empty vector was set at a threshold of +/- 1.5 log2 fold change and a p-value with false discovery rate of less than 0.05. For the EAPP mutant, the p-value did not include false discovery rate thresholding due to the limited experimental effects of this mutant. **(D)** 75 genes were significantly regulated by KLF11 overexpression (p < 0.05) and are directly bound by KLF11 as determined by chromatin immunoprecipitation. Examination of effects of the three chromatin decoupling mutants reveals that expression is frequently altered in the presence of one or more of these variants.

Recent studies support a model whereby KLF11 functions by binding to GC-rich sites within promoters of different gene networks involved in the regulation of metabolism and cell growth [2-4,7-12]. This data is in agreement with the biological role of this transcription factor in cancer and diabetes. However, how binding of KLF11 to these promoters regulates gene expression remains to be fully understood. Recent data demonstrate that the KLF11 protein behaves as a scaffold for recruiting different chromatin cofactors via distinct structural motifs (Figure 1A). The Sin3 Interacting Domain (SID) between amino acids 22–40 enables coupling of KLF11 to the Sin3/HDAC system [11-19]. Introduction of proline residues at amino acids 29–30 interrupts the association between KLF11 and the Sin3 scaffold protein, which includes interactions with HDAC1/2. The region between 281 and 373 amino acids is a proline-rich domain that couples with a variety of WD40 proteins, including G-protein coupled receptors [4]. The A347S mutation observed in MODY7 (neonatal diabetes) falls within this domain and has been demonstrated to decouple KLF11 from novel transcription factor Gβ2. The zinc finger domain has been demonstrated to interact with histone acetyltransferases p300 and CBP [3,20,21]. Finally, the region between 483 and 487 dictates interaction between KLF11 and HP1α [7]. Deletion of this C-terminal portion decouples HP1α from histone methyltransferases SUV39 and G9a, leading to defects in metabolism and tumor suppression. Currently, it is unknown whether these systems work in isolation or in a cooperative manner to regulate gene expression.

To address this question, we first preformed genome-wide promoter binding analysis of wild type KLF11. We then limited our analysis to 5 kb upstream and downstream of the transcription start site of known gene promoters. Examination of the distance of KLF11-bound peaks from each TSS revealed that the majority (~60%) was within 500–1000 base pairs of the TSS (Figure 1B). Gene ontological analysis of KLF11-bound targets reveals association to a large number of metabolic processes, including many already characterized as regulated by KLF11, such as insulin regulation [3,4,12] and Akt/TOR signaling [11] (Figure 1C). Next, we performed genome-wide expression profiles using mutants for these sites which have been shown to specifically uncouple KLF11 from each of these chromatin remodeling proteins, namely EAPP (Sin3/HDAC), A347S (WD40 proteins), and Δ486 (HP1α/HMT) (Additional file 1: Figure S1). Of the 404 gene targets identified by chromatin immunoprecipitation, for which corresponding gene expression data was available, 19% (n = 75) display alteration in the presence of the wild type KLF11 (p < 0.05). Clustering analysis reveals that the expression pattern of these gene targets is disrupted in the presence of one or all of the described mutants. Thus, we conclude that KLF11-mediated gene expression may be disrupted by decoupling of the transcription factor from its chromatin co-factors in a singular or combinatorial fashion.

### Distinct gene networks are regulated by the combinatorial coupling of KLF11 to chromatin partners

**Figure 2:**
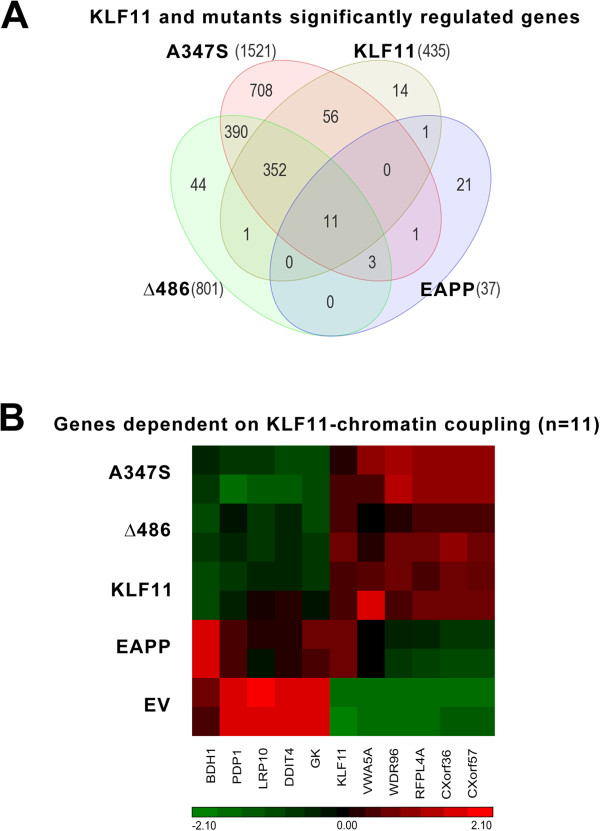
**KLF11 differentially couples to distinct chromatin co-factor systems. (A)** Venn diagram of the overlap of genes significantly regulated by wild type KLF11 and its mutants. Genes that occur at the overlap of KLF11 and all three of its chromatin-coupling mutants were deemed as independent of the effects of chromatin de-coupling and therefore inherent to the protein itself. **(B)** Heatmap of the 11 KLF11-regulated genes that are dependent on interaction with all three chromatin coupling systems.

**Figure 3:**
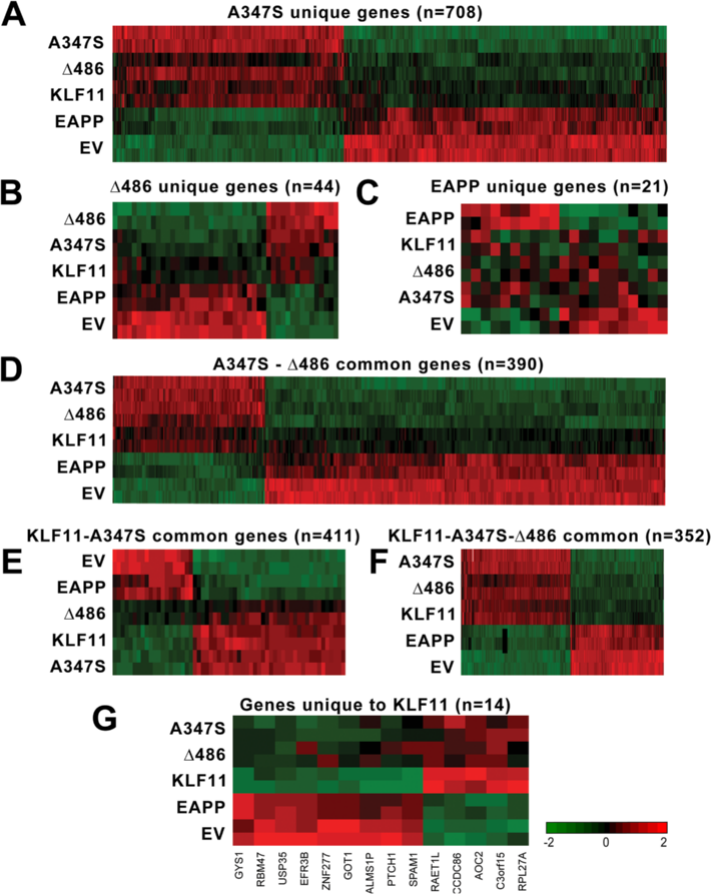
**KLF11 regulates gene expression through combinatorial and singular chromatin coupling events. (A)** The A347S mutant, which decouples KLF11 from WD40 proteins, displays the most genome wide effects, uniquely affecting the expression of 708 genes, whereas the **(B)** Δ486 deletion mutation (decouples KLF11 from HP1/histone methyltransferases) and the **(C)** EAPP mutation (decouples KLF11 from binding the Sin3 scaffold protein and subsequently histone deacetylases) uniquely regulate only 44 and 21 genes, respectively. **(D)** Examination of the overlap between genes significantly regulated by the A347S mutant reveals that approximately 50% are regulated in a similar fashion to the Δ486 mutant, although with varying degrees of intensity. The EAPP mutant, however, displays near complete reversal of these targets. **(E)** Wild type KLF11 and the A347S mutant share 411 targets apart from either the EAPP or Δ486 mutants, although **(F)** 352 targets exists that are shared between all three systems. These data indicate that KLF11 chromatin coupling occurs in a largely combinatorial fashion. **(G)** Only 14 genes were identified that are uniquely regulated by KLF11, independent of chromatin coupling to the transcription factor. Ontological analysis of these genes reveals roles in cancer, cellular proliferation, and metabolism.

Next, we investigated which gene targets are uniquely regulated by each of these pathways. Statistical analyses of each expression profile show that, indeed, when compared with the empty vector control condition, each mutation induces changes in gene expression that are either specific to each chromatin system or similar to the wild type. Thus, comparison of the genes significantly affected by each condition (p-value <0.05 and fold change ± 1.5) shows that 97% of transcripts that are modulated by the wild type KLF11 are also regulated by any of the three mutations, with the A347S and Δ486 mutations sharing the largest number of genes with the wild type (Figure 2A). In fact, of the 801 genes modulated by the Δ486 mutation, 93.9% are also modulated by the A347S mutation. Finally, the three mutants share with KLF11 only 11 different genes, which likely require an intact coupling of this transcription factor to all the chromatin proteins involved in its function (Figure 2B). We observe that the A347S mutation shows the largest number of significantly regulated transcripts (n = 708) upon the decoupling of KLF11 from WD40 proteins (Figure 3A). By contrast, the subsets of genes uniquely regulated by the Δ486 or EAPP mutants are much smaller, 44 and 21 genes, respectively (Figures 3B-C).The Δ486 mutation uniquely modulates only 44 genes (5.5%) of the 801 genes significantly modulated under expression of the mutant. Almost half of all Δ486 modulated genes (n = 390) are common to the A347S mutant (Figure 3D) and the other half are common to both A347S and wild type KLF11 (Figure 3E). Interestingly, the Δ486 and the A347S mutants have, in general, a similar direction of modulation, although with varying degrees of signal intensity. On the other hand, the A347S and Δ486 modulated genes are almost always completely reverted by the EAPP mutation, in which the signals are constantly proximal to empty vector values.Under the same conditions, the A347S mutant, which associates to the development of human juvenile diabetes, induces changes in 1521 genes. 46.5% of the modulated genes (708/1521) are specific to this mutation, while approximately half of the genes (48%) are common to the Δ486 mutant and 43.3% are common to both A347S and wild type KLF11 (352/423, Figure 3F). Finally, 7% of the genes (n = 50) are common to wild type KLF11, and only 15 common to the EAPP mutant. The EAPP mutation, which disrupts coupling to the Sin3-HDAC complex, reverses the expression of most genes induced by the wild type KLF11 or by the other two mutations.

### Identification of KLF11-regulated genes for which expression is independent of known chromatin coupling events

Subsequently, we examined genes for which expression might be regulated by KLF11 in a manner that does not involve the chromatin remodeling machines examined above. A small percentage of genes are still significantly modulated uniquely by wild type KLF11 (Figure 3G). Ontological classification demonstrates that these 14 genes are associated with diabetes (ALMS1P; RAET1L; GYS1; RBM47; EFR3B), cancer (RPL27A; SPAM1; PTCH1; ZNF277; USP35; CDC86), or metabolism (C3orf15; AOC2; GOT1). Taken together, these data indicate that the vast majority of KLF11’s biological processes are dependent on its interaction with chromatin regulators within our model system. A repertoire of chromatin-independent function is predicted, as the transcription factor is capable of interaction with gene promoters directly. Alternatively, the regulation of these genes may involve yet unidentified chromatin co-factor.

### Pathway reconstruction of combinatorial KLF11-regulated chromatin pathways by ontological approach

**Figure 4:**
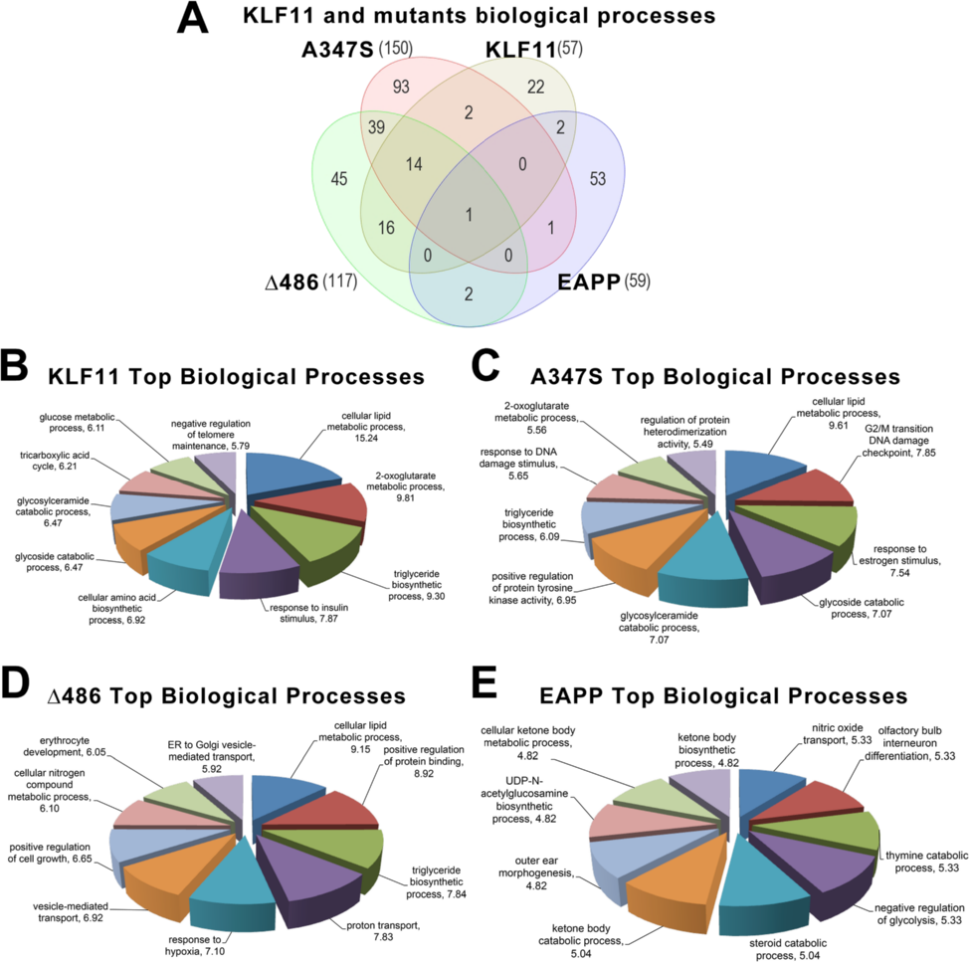
**Biological regulated by KLF11 and its chromatin binding partners.** Genes significantly regulated by wild type KLF11 and its mutants compared to empty vector were analyzed for enrichment of biological processes by an ontological approach. A threshold of 3 genes and a p-value of less than 0.05 by Fisher’s Exact Test were required to be considered significantly regulated by the transcription factor and its mutants. **(A)** Venn diagram of biological processes demonstrates that the A347S mutant causes the largest number of genome-wide effects with significant alterations in 150 processes, 93 of which are unique to the mutant. Only one biological process, lipid cellular metabolism, is dependent on the combinatorial effects of all three chromatin coupling systems. The top biological processes were computed for each of the proteins compared to empty vector. The pie charts for **(B)** Wild type KLF11, **(C)** A347S mutant, **(D)** Δ486 mutant, and **(E)** EAPP mutant display the top ten scoring biological processes for each with enrichment percentage displayed. These results indicate that the effects of KLF11 coupling to chromatin co-factors impact on a large number of biological functions.

Gene Ontology (GO) enrichment analysis of the genes modulated by wild type KLF11 and each of the three mutations show various common biological processes (Figure 4A). Indeed, the wild type shares 60% (34/57) of the biological processes with at least one of the mutants. One of the biological processes, lipid cellular metabolic process, is enriched in wild type KLF11 and all three mutants (Additional file 2: Table S1). Among the most highly enriched biological processes, 14 terms are common to wild type KLF11 and the A347S and Δ486 mutations. Variations of the intensity of signal, the number of genes implicated, and the score of GO enrichment for these 14 biological processes are shown in Additional file 2: Table S1. Interestingly, wild type KLF11 and mutants conserve several biological processes related to anabolism or catabolism of lipids, amino acids, and glycoconjugates. Other common groups include redox processes of protein homodimerization, regulation of the activity of protein kinases, and response to the estrogen.

Two of the wild type KLF11 biological processes, the G2/M transition during DNA damage checkpoint and cilium assembly, are also conserved in the A347S mutation. There are 16 biological processes exclusively conserved between wild type KLF11 and the Δ486 mutant. These include cellular metabolic processes, such as aldehyde and nitrogen compound metabolism, isocitrate and 2-oxoglutarate metabolism, glycosaminoglycan catabolic processes, and fatty acid beta-oxidation.

Interestingly, the A347S and Δ486 mutations share more than a quarter of biological processes (n = 39) that are absent in wild type KLF11 and the EAPP mutant. Between these 39 GO terms, we found enrichment of terms associated to the monitoring of the DNA transcription, RNA polymerase I transcription promoter regulation, and epigenetic control of gene expression, such as histone H3 acetylation, chromatin remodeling, protein acetylation and deacetylation, and regulation of phosphorylation. Wild type KLF11 and EAPP share only two GO-terms in common: branched chain amino acid family catabolic processes and negative regulation of TOR and signaling cascade.

Overall, 22 of 57 (38.6%) of the biological processes enriched in the wild type KLF11 are not present any of the three mutants. Among the 22 most enriched biological process found only in the wild type KLF11, we observed several metabolic processes, such as glucose, carbohydrate, and very long-chain fatty acid metabolism, oxaloacetate metabolism, fatty acid homeostasis, and oligosaccharide metabolism. Top processes associated with wild type KLF11 expression are shown in Figure 4B and Additional file 3: Table S2. Other specific mechanisms of wild type KLF11 lost by mutation are two mechanisms associated with gene expression (transcription from the RNA polymerase III promoter and mRNA capping) and two biological processes associated with the integrity of the DNA (the DNA repair and the response to DNA damage stimulus). Finally, three important biological processes (e.g., the mitotic cell cycle, the negative regulation of insulin-like growth factor receptor signaling pathway, and the negative regulation of epithelial cell proliferation) are also lost by the mutants. From these data, we conclude that the combinatorial association between the transcription factor and a variety of chromatin coupling systems dictates the majority of KLF11 function, particularly in regulating gene expression and metabolism. The high degree of overlap suggests a form of “epigenetic redundancy” to ensure the proper regulation of these gene targets and processes.

### Pathway reconstruction of singular KLF11-regulated chromatin pathways by ontological approach

Of the mutants, A347S displays the highest number of unique biological process that are not enriched in the wild type (n = 93). Top processes associated with A347S mutant expression are shown in Figure 4C and Additional file 3: Table S2. Interestingly, almost 30% of these biological processes are associated with the structure and maintenance of DNA or telomeres (Additional file 4: Table S3, List 1), processes related to phosphorylation and dephosphorylation, deacetylation, methylation, and demethylation (Additional file 4: Table S3, List 2), and processes of transcriptional regulation by poly-II and mRNA transport (Additional file 4: Table S3, List 3).

In turn, the Δ486 mutant exclusively enriches 45 biological processes that are mediated by the wild type KLF11 or in other mutants. Top processes associated with Δ486 expression are shown in Figure 4C Additional file 3: Table S2. Unlike the A347S mutant, mutation Δ486 is particularly enriched in processes associated with metabolic and biosynthetic processes, protein export–import, and energy protein modification associated processes (Additional file 5: Table S4, List 1). Other groups of processes specifically enriched in this mutant are associated with proliferation and epithelial cell survival (Additional file 5: Table S4, List 2).

The EAPP mutant did not display robust gene expression changes within the experiment. However, utilizing a more permissive cutoff yields a list of 53 biological categories, although each only contains a single gene. We find enrichment of biological processes involving nitric oxide transport, catabolic processes, and developmental morphogenesis function, among others. Top processes associated with EAPP expression are shown in Figure 4E and Additional file 3: Table S2. From these data, we are able to identify singular KLF11 chromatin coupling events dictating a number of critical processes, particularly in management of cell cycle control and DNA replication, ones that inherently require a more specific and finely regulated degree of control to execute their programs under strict spatial and temporal constraints.

### Identification of canonical signaling pathways mediated by KLF11 and mutants

**Figure 5:**
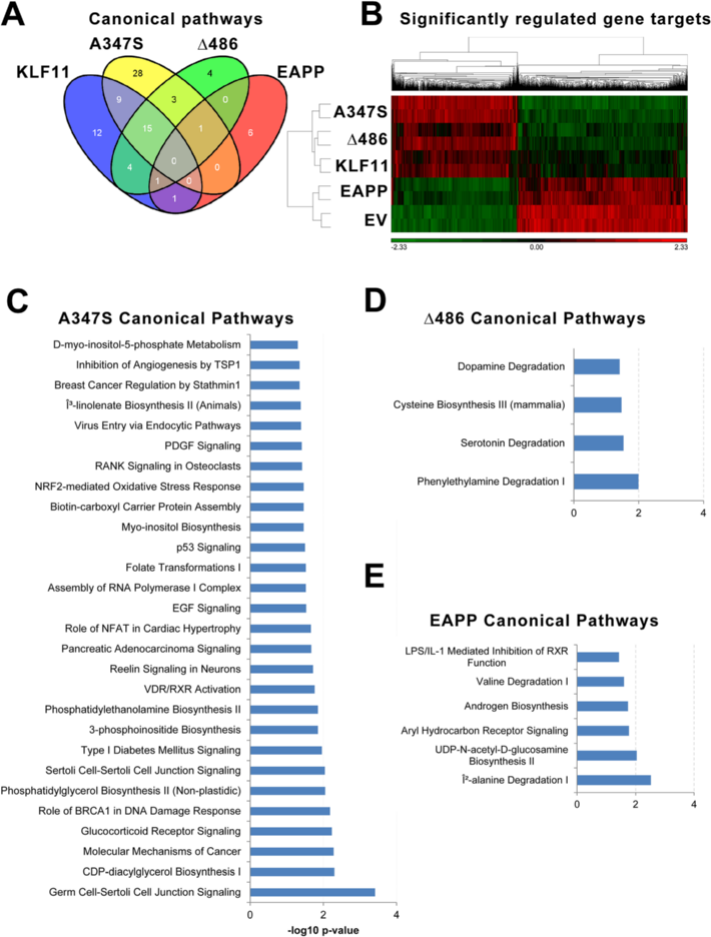
**Canonical signaling pathways mediated by KLF11 and mutants.** Using the Ingenuity Global Canonical Pathways algorithm, significantly modulated signaling pathways were identified for wild type KLF11 and its mutants. A p-value of less than 0.05 as determined by Fisher’s Exact Test was used as criteria for significant association of focus genes to predetermined pathways curated from published literature. **(A)** Venn diagram of significant canonical pathways regulated by wild type KLF11 and mutants reveals that the effects of the EAPP mutation are less significant in this experiment compared to the A347S and 486 mutants. These data are supported by the clustering of all significantly regulated targets as shown in **(B)** which demonstrated that the A347S and Δ486 mutants cluster closely with wild type KLF11 while the EAPP mutant clusters with the empty vector control, indicating complete reversal of the effects of the other proteins. **(C)** Among the 28 top scoring unique canonical pathways mediated by the 374 mutant are signaling pathways, including, EGF, PDGR, p53, RANK and reelin signaling, and glucocorticoid receptor signaling. The Δ486 **(D)** and EAPP **(E)** mutants mediate 4 and 6 pathways respectively, both of which are centered on processes involved on the degradation of biogenic amines. These data support KLF11 as a master transcriptional regulator that mediates large, interconnected signaling cascades.

Subsequently, we wished to further assess the relatedness between the gene targets significantly modulated by KLF11 and its mutants utilizing a semantic-based approach and the Ingenuity Global Canonical Pathways (GPC) algorithm. The input dataset of significantly regulated gene targets was compared against the IPA canonical pathways, which are curated from published literature. The significance of the associations between the data set and a given canonical pathway is determined by the ratio of the number of genes mapping to the pathway divided by the total number of pathway genes. A p-value is calculated using Fischer's Exact Test determining the probability that the association between the data set and the pathway occurs by chance alone. GPC analysis of genes modulated by the wild type KLF11 and the three mutants shows a variety of significantly enriched pathways (p-value <0.05). The wild type KLF11 shares about 71% (30/42) of its pathways with at least one of the mutants (Figure 5A). The most important intersection of common pathways is observed between wild type KLF11 and mutants A347S and Δ486. Indeed, 90% (38/42) of these pathways are also enriched in at least one of these two mutants. Only two pathways are shared between the wild type and the EAPP mutant.Nevertheless, all genes implicated in these pathways are differently modulated by wild type KLF11 and each one of the mutants. The expression of these genes significantly regulated by wild type KLF11 and the A347S and Δ486 mutants are almost always modulated in the same sense and clustered together (Figure 5B). In contrast, the EAPP site produces a complete reversion of the signals with the levels of gene expression close to the empty vector level, as evidence by its clustering with empty vector apart from the wild type KLF11 and other mutants.

Twelve of the 42 significantly enriched pathways are conserved only in the wild type KLF11. These normal functions of the wild type KLF11 are potentially lost by the three studied mutations. The most enriched of these pathways includes the nucleotide excision repair pathway and the alpha-adrenergic signaling pathways. Interestingly, of all the remaining pathways, 80% (9/11) are metabolic biosynthetic pathways.Mutant A347S possesses the most exclusive pathways. Indeed, 28 pathways are enriched in the A347S mutant compared to wild type KLF11 and other mutants (Figure 5C). Interestingly, over 30% (9/28) of these pathways are signaling pathways, including, EGF, PDGR, p53, RANK and reelin signaling, and glucocorticoid receptor signaling. Furthermore, pathways involved in diabetes and pancreatic adenocarcinoma are also present. These pathways may explain the massive number of genes differentially modulated by this mutation (n = 1521 genes) and suggests that this mutant could induce new potentially unexpected and significant physiopathological changes.86% of the enriched pathways in the Δ486 mutant are shared with the wild type KLF11 or the other mutants. Only four new pathways are specifically enriched by the Δ486 mutation (Figure 5D). Interestingly, these pathways are implicated in the degradation of biogenic amines (an important field associated to physiopathology of depression and metabolism of drugs of abuse). Finally, the EAPP mutant influences only six pathways exclusively (Figure 5E), also primarily related to the degradation of biogenic amines. Taken together, we conclude that KLF11 chromatin-coupled gene targets delineate into specific, well-ordered pathways, consistent with KLF11’s position as a master transcriptional regulator that mediates large cascades of other transcription factors and regulators. The effects of KLF11 chromatin coupling are therefore not incidental to its function but a primary means by which to execute gene activation or repression.

### Reconstruction of downstream biological and disease networks mediated by KLF11 and mutants

**Figure 6:**
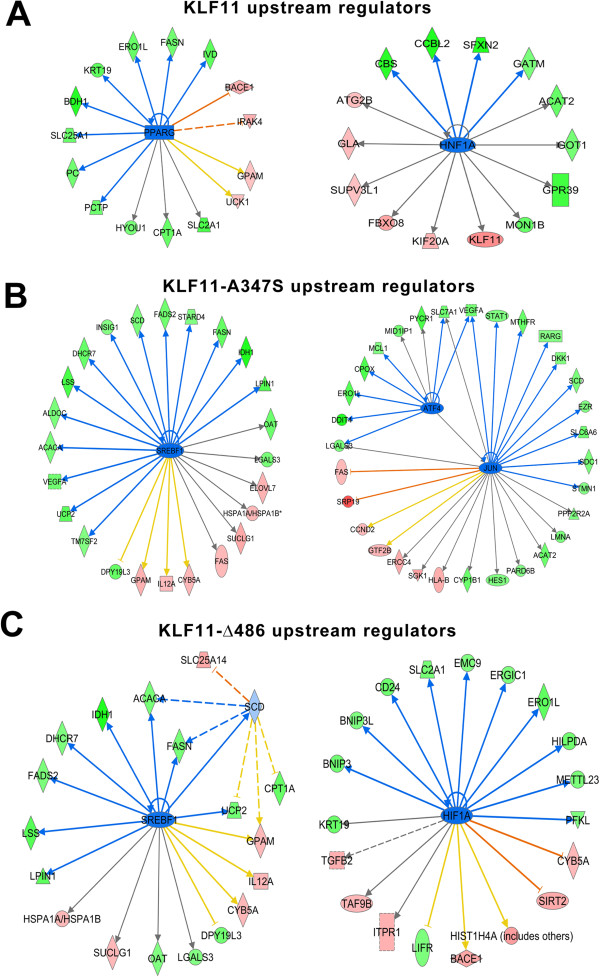
**Biological disease networks mediated by KLF11 and its mutants.** Using Ingenuity’s network analysis tool, significantly regulated genes by KLF11 **(A)** and its A347S **(B)** and Δ486 **(C)** mutants were examined for connectivity. Networks were scored by relevance and a p-value assigned by Fisher’s Exact Test to discriminate networks generated by chance alone. A threshold of p < 0.05 was employed to determine significant networks. Networks were then assessed for association to known biological or disease processes. Wild type KLF11 and its mutants share three processes: death and survival, hereditary disorders, and molecular transport. Of the identified processes, 10 are unique to wild type KLF11 and 6 and 8 are unique to the A347S and Δ486 mutants, respectively. A number of known KLF11-mediated biological and diseases processes were revealed by this analysis, providing an internal control, including endocrine disorders, gastrointestinal disorders, and cellular growth and proliferation processes. However, the analysis also generated a number of novel biological and disease processes that remain to be experimentally validated for KLF11.

We next explored the degree of connectivity between KLF11 and mutant-regulated genes and their pathobiological associations. Networks of significantly regulated genes were algorithmically generated based on their connectivity and assigned a score that encapsulates the relevance of the generated network to the original list of focus genes. All edges are supported by at least one literature reference of direct physical, transcriptional, and enzymatic interactions, or from canonical information stored in the Ingenuity Pathways Knowledge Base. A right-tailed Fisher’s Exact Test was used to calculate the p-value for networks and a threshold of p < 0.05 used to determine significance. A functional analysis of a network then determined the biological functions and/or diseases that are most significant to the genes contained within the network (Figure 6).

Of the identified processes, only three were common to wild type KLF11 and all three mutants: cell death and survival, hereditary disorders, and molecular transport mechanisms. Wild type KLF11 possessed the most unique networks (n = 10) and included a number of characterized biological and disease networks, including cellular growth and proliferation [22], endocrine disorders, reproductive system development and function [23], and biliary hyperplasia, among others. The A347S mutant uniquely associates to 6 networks, including ones linked to auditory disease, cellular compromise, and organismal development. The Δ486 mutant uniquely associated to 8 networks. Among the Δ486 mediated networks are significant associations to gastrointestinal disease, connective tissue development, and immune presentation and response. Together, these data recapitulate the repertoire of known KLF11-mediated functions and uncover a number of new potential functional and disease associations for future studies.

### Identification of upstream regulators of KLF11-chromatin coupled pathways

Finally, we wished to identify the upstream transcriptional regulators using Ingenuity’s Upstream Regulator (UR) analysis. The UR analytic is based on prior knowledge of the expected effects of transcriptional regulators and their target genes stored in the Ingenuity Public Knowledge Base. The algorithm examines how many known targets of each transcriptional regulator are present in the list of significantly regulated targets and compares direction of expression to that expected from previously published data. For each potential regulator, an overlap p-value using Fisher’s Exact Test and an activation z-score was computed, the latter of which determines the activation state. A threshold p-value of <0.01 and activation score of +/- 2 was considered significant for our purposes. The end result was the identification of upstream regulators of KLF11 or its mutants that permit the generation of plausible signaling cascades mediated by these upstream regulators through KLF11 chromatin coupling.

**Figure 7:**
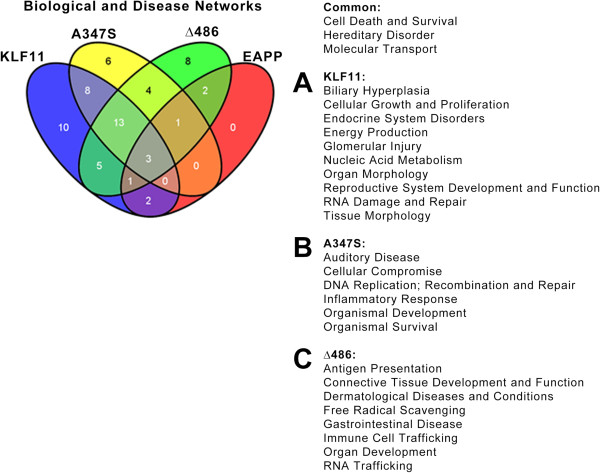
**Upstream regulators of KLF11 and its mutants.** Gene targets significantly regulated by KLF11 and its mutants were analyzed by Ingenuity’s Upstream Regulator Analytic that compares the experimentally derive activation or inhibition of focus molecules to relationships between upstream regulators and target molecules known from published data. For each potential upstream regulator and its targets, a p-value for the degree of overlap between that of the protein under study and a z-score for the activation status are calculated. For our study, a threshold of p < 0.05 and a minimum z-score of +/- 2 were employed. The full results are published in Additional file 6: Table S5 and sample signaling cascades presented for wild type KLF11 **(A)**, A347S **(B)**, and Δ486 **(C)**. No cascades were generated that proved significant for the EAPP mutant although a more permissive criteria of a p-value of less than 0.05 did generate a number of single gene associations listed in Additional file 6: Table S5, including a variety of other KLF proteins, hinting at co-regulation within the KLF family. Many of the upstream regulators, such as PPARG, are previously identified co-regulators of KLF11. While the upstream regulators frequently overlap between wild type and mutant conditions, the end gene targets are unique to each condition. These results suggest that the deregulation of KLF11 from a large number of gene networks is completely dependent on the effective coupling of the transcription factor to its chromatin co-factor system.

The results of the UR analysis are presented in Additional file 6: Table S5. Wild type KLF11 displayed two significant upstream inhibitory regulators, PPARG and HNF1A, both of which have previously been implicated in the regulation of the transcription factor or as a co-regulator (Figure 7A) [24]. The A347S mutant, consistent with the gene level, pathway, and network analyses, is the most disruptive, possessing 9 activating upstream regulators and 23 inhibitory upstream regulators. Interestingly, EZH2, a histone methyltransferases, is identified as potential upstream activator. Previous reports have identified an antagonistic relationship between KLF10 and the functions of EZH2/Polycomb Repressive Complex [21]. This data hints to other important chromatin couplings to KLF11 that remain to be completely elucidated. Figure 7B shows the genes affected in the presence of the mutant when 486 is inhibited by SREBF1 or JUN and ATF4. For the 486 mutant, 6 upstream activators were identified and 15 upstream inhibitors, including SREBF2 and HIF1A (Figure 7C). Although the EAPP did not display any significant upstream regulators as defined by our full criteria, a number of other KLF proteins (KLF1, 3, 7, 13) were identified as upstream regulators by p-value alone, suggesting that inter-association of KLF proteins is also critical to KLF11 function. In addition, we find that KLF10 is identified as affected downstream of both the 486 and A347S mutants through multiple KLF11 upstream regulators. Together, these data suggest that transregulation among KLF proteins is critical to their function and reveal the potential for a chromatin-dependent interplay between family members, permitting additional specificity in the regulation of distinct nodes within their targeted gene expression networks. Although a number of the regulators are common to the wild type KLF11 and the A347S and Δ486 mutants, indicating that mutation does not necessarily impair upstream regulation or co-regulation, the activation or inhibition of these proteins regulates a distinct subset of endpoint genes. The absence of wild type KLF11 targets and acquisition of new, inappropriate targets in the presence of mutation places KLF11 chromatin coupling events at the center of the transcription factor’s function or dysfunction.

In summary, here we have identified the precise interplay between KLF11 and three of its chromatin coupling systems, namely Sin3/HDAC, HP1/HMTs, and WD40 proteins, on a genome-wide scale. The functional repertoire of KLF11 appears to be dictated by both combinatorial effects between these systems and singular chromatin coupling events. Redundancy, particularly in critical biological processes such as metabolism and organismal development, occurs between systems to ensure preservation of critical functions. On the other hand, singular chromatin coupling events exist to ensure precision in processes requiring strict spatial and temporal parameters to avoid inappropriate signaling.

## Discussion

Until recently, the relationship between chromatin and transcription factors appeared largely passive. However, the evidence that overexpression of four transcription factors—Oct4, Sox2, m-Myc, and KLF4—is sufficient to reprogram fully differentiated fibroblasts into pluripotent ES-like cells suggests an active role for transcription factors in the epigenetic regulation of chromatin state [25]. KLF proteins, in particular, have demonstrated a multitude of feed-forward effects on epigenetic regulation of gene expression. Here, we propose KLF11 as a model for understanding the critical role of sequence-specific factors in epigenetic regulation of a multitude of pathobiological processes.

KLF proteins deliver epigenetic information to gene promoters through three primary mechanisms: (i) sensing and translating environmental stimuli into a program of gene expression, (ii) sequence-specific targeting of chromatin remodeling complexes to gene promoters, and (iii) transactivation of other transcription factors to assist in the regulation of large networks of interdependent genes. Transient regulation of gene expression occurs through association with histone acetyltransferases (HATs) and histone deacetyltransferases (HDACs), while long-term gene silencing is enacted by interactions with histone methyltransferases (HMTs) and DNA methyltransferases (DNMTs). No single classification scheme may accurately characterize the function of KLF11 in its entirety. However, the classification of family members by virtue of its co-factors provides a framework to understand the functional differences in the manner in which it engages chromatin to activate or repress transcription in a dynamic and reversible manner.

The association between KLF11 and chromatin coupling is well established. Initial biochemical characterization of KLF11 revealed that its N-terminus possesses a domain that adopts an α-helical conformation and that mutations within this domain significantly disrupted its binding to Sin3 and the subsequent recruitment of HDACs [26,27]. Subsequently, KLF11 was found to associate with HP1α [7], one of the epigenetic “gatekeepers” of gene silencing through its extreme C-terminus [28-30]. HP1 proteins repress gene expression by binding to H3K9me marks and interacting to H3K9 histone methyltransferases, such as G9a or SUV39H1, which methylate this same residue on adjacent nucleosomes [31,32]. Deletion of the C-terminus leads to deregulation of tumor suppression functions mediated by KLF11. Finally, KLF11 has also been demonstrated to associate with WD40, WWI, WWII, and SH3-domain containing proteins through a proline-rich domain [4]. Interaction of KLF11 and WD40 protein Gβ2, for example, is disrupted in the presence of the A347S mutation, the variant associated with development of Maturity Onset Diabetes of the Young 7 (MODY7). The effect of decoupling of KLF11 to chromatin co-factors, however, has only been studied on small subsets of genes related to the system of focus.

The investigation presented here is the first to attempt to dissect the roles of KLF11 and chromatin coupling on a genome-wide level to ascertain the combinatorial or unique effects of each type of previously identified interacting chromatin system. Utilizing a single cell type with overexpression of wild type KLF11 and the three previously described mutants, A347S, Δ486, and EAPP, we were able to examine the interplay between coupling of the transcription factor to the WD40, HP1/HMT, and Sin3/HDAC systems, respectively. By introducing saturating amounts of each mutant into pancreatic epithelial cells, we were able to tease apart the relative and overlapping contributions of each chromatin coupling mechanism to the regulation of pathobiological gene networks. Our results reveal that in the pancreatic epithelial cell utilized as our model, a large number of the affected genes, networks, and signaling cascades are modulated by the coupling of KLF11 to its variety of chromatin systems. The number of genes identified as regulated independently of these systems represented <1% of the total genes mediated in the presence of wild type or mutant proteins.

It is important to underscore that the study reported here is unique as it uses genome-wide approaches to dissect the function of several different chromatin-based pathways in the transcriptional function of a single KLF protein. The KLF family of transcription factors consists of 17 members [1]. Sequence identity at the carboxyl terminus among KLF family members is greater than >65%, suggesting the regulation of similar types of gene promoters and the potential for synergistic or antagonistic regulation by family members. Despite the high degree of sequence identity in their DNA-binding ability, the functional activities of KLF members differ widely due to the high degree of variability within the N-terminal domain of each protein, the region responsible for interaction with chromatin machinery. Although the highly conserved DNA-binding domain denotes their familial origins, the ultimate functional identity of each KLF protein rests in its their ability to couple to the molecular machinery that regulate gene expression, in particular to bind writers, readers, and erasers of the histone code. Although some family members bind to the same chromatin partners as KLF11 [33], the degree to which the gene networks and biological processes identified here may be generalized to other family members remains to be elucidated. However, it is tempting to speculate that the most closely related proteins KLF9, KLF10, KLF11, KLF13, KLF14, and KLF16 being extensively studied in our laboratory because of their participation in metabolic processes, may at least regulate some overlapping gene networks. These proteins contain distinct motifs which couple to similar and also distinct chromatin remodeling complexes. In fact, it has been shown that these proteins couple to the same chromatin regulators in organisms ranging from flies to human. Notably, disruption of the KLF11 orthologous fly gene Cabut, leads to metabolic problems [34]. In human, alterations in KLF11 causes neonatal and juvenile Diabetes while its closely related gene, KLF14 causes obesity and adult type II diabetes [35]. Interestingly, this function of KLF14 appears to be influenced by a cross regulation with KLF13. This is interesting since our microarray data shows that, KLF9 is significantly downregulated by wild type KLF11 and KLF10 is upregulated by the 486 and A347S mutants. Therefore, this data suggests that this subgroup of highly related subfamily of proteins, which appears to have evolved from the fly ancestor Cabut, share similar chromatin coupling mechanisms, similar general function in metabolism, and may, to some extent, share targeted gene networks However, they still have some degree of biochemical differences as well as phenotypic outcomes. On the other hand, the rest of the KLF proteins, which are structurally and functionally less related to KLF11 but may still share some chromatin partners, are predicted to have fewer overlapping co-regulated gene networks. In this regard, we are optimistic that the type of investigations reported here will fuel the interest of both basic biologists and translational scientists who are keen on better understanding the function of this extended family of transcription factor proteins as it relates to biochemistry, genetics, epigenetics, general biology and the pathobiology of human diseases.

## Conclusion

More than fifteen years ago, our research team hypothesized that the discovery and study of KLF proteins and their chromatin cofactors would assist in unraveling complex human diseases. In the intervening years, our understanding of the interplay between KLF proteins and epigenetic machinery in transient and long-term gene regulation has grown exponentially. With knowledge inferred from the predictive power of rationally derived computational models, we propose a new paradigm for KLF regulation of gene networks through the translation of input from cellular milieu into epigenetic information to affect changes in chromatin structure. KLF proteins currently provide the best model for understanding the interactions between mechanisms of “hard inheritance” (environmental and genetic variation) and “soft inheritance” (epigenetic variation) in underscoring the phenotypic variability observed in complex disease mechanisms. Perhaps most importantly, these new pathways open up infinite possibilities for targeting new molecules and processes in therapeutic intervention and disease management.

## Methods

### Cell culture

Panc1 cell lines were obtained from the American Type Culture Collection (ATCC, Rockville, MD). Cells were cultured as described previously [4,36].

### Constructs

Standard molecular biology techniques were used to clone full length KLF11, KLF11-A347S, KLF11-EAPP, and KLF11-486 into pcDNA3.1/His (Invitrogen, Carlsbad, CA). All constructs were verified by sequencing at the Mayo Clinic Molecular Biology Core Facility. Epitope-tagged (6XHis-Xpress™) KLF11, KLF11-A347S, KLF11-EAPP, KLF11Δ486 variants as well as empty vector (Ad5CMV) were generated as recombinant adenovirus in collaboration with the Gene Transfer Vector Core at the University of Iowa.

### Genome-wide expression profiles of KLF11 and mutants

Panc1 epithelial cells were plated at a density of 1×10^6^ cells/100 mm dish and transduced with empty vector, KLF11, KLF11-A347S, KLF11-Δ486, or KLF11-EAPP at an MOI of 150. Relative expression levels were confirmed by Western Blot (Additional file 1: Figure S1). RNA was prepared as previously described from pooled biological triplicates [37]. Global gene expression profiling was carried out in technical duplicate at the Microarrays Facility of the Research Center of Laval University CRCHUL utilizing the Affymetrix Human Gene 1.0 ST arrays (28,869 well-annotated genes and 764,885 distinct probes). Intensity files were generated by Affymetrix GCS 3000 7G and the Gene-Chip Operating Software (Affymetrix, Santa Clara, CA). A subset of genes was validated by qPCR as previously described (Additional file 7: Figure S2) [20,38].

### Genome-wide promoter binding profile of KLF11

Panc1 epithelial cells were transfected with full-length His-tagged KLF11. ChIP was performed as previously described [38-41] using an antibody against the His-Tag (OMNI D8; Santa Cruz Biotechnology) to detect recombinant expression of KLF11. Non-specific IgG antibody was utilized as a negative control. Binding activity was derived using the NimbleGen human promoter hybridization system (Madison, WI). Peaks were detected by searching for >4 probes where signals were above the specified cutoff values (90% to 15%) using a 500 bp sliding window along 5 kb upstream and downstream of the transcriptional start site in human promoters. Each peak was assigned a score that is the log2 ratio of the fourth highest probe in each peak. If multiple peaks are present, the peak nearest the TSS is reported. Ratio data was then randomized 20 times to evaluate the false discovery rate (FDR). Only peaks with FDR scores <0.2 were deemed high confidence binding sites and reported.

### Data analysis

Data analysis, background subtraction and intensity normalization was performed using Robust Multiarray Analysis (RMA) [42]. Genes that were differentially expressed along with false discovery rate (FDR) were estimated from *t* test (>0.005) and corrected using Bayes approach [43,44]. A threshold of +/- 1.5 log2 fold change with a p-value with FDR of less than 0.05 (without FDR for EAPP mutant) was used to determine significantly regulated targets. Data analysis, hierarchical clustering, and ontology were performed with the OneChanelGUI to extend affylmGUI graphical interface capabilities and Partek Genomics Suite, version 6.6 (Partek Inc., St. Louis, MO) with ANOVA and GO ontological analysis [45]. Selected probes and their fold changes were loaded into Ingenuity Pathways Analysis Software (Ingenuity® Systems, http://www.ingenuity.com) for annotation, redundancy checks, canonical pathway, biological network, and upstream regulator analysis using default parameters.

### Availability of supporting data

The data sets supporting the results of this article are available in the NCBI repository, GSE56778.

## Competing interests

The authors declare that no competing interests exist.

## Authors’ contributions

EC, AG, GL, and AM carried out the molecular, promoter binding, and gene expression studies, participated in the bioinformatics analysis, and drafted the manuscript. JI and RU conceived of the study, participated in its design and coordination, and helped to draft the manuscript. All authors read and approved the final manuscript.

## Supplementary Material

Additional file 1: Figure S1 Analysis of wild type KLF11 and mutant protein expression. Protein expression of epitope-tagged (His_6_-Xpress™) wild type KLF11 and the A347S, Δ486, and EAPP mutants demonstrating similar overexpression in Panc1 cells. α-Tubulin was used as a loading control. Click here for file

Additional file 2: Table S1 Overlapping biological function mediated by KLF11 and mutants. Click here for file

Additional file 3: Table S2 Top biological processes mediated by KLF11 and mutants compared to empty vector. Click here for file

Additional file 4: Table S3 Biological processes unique to the decoupling of KLF11 from WD40 proteins (A347S mutant). Click here for file

Additional file 5: Table S4 Biological processes unique to the decoupling of KLF11 from HP1a/HMT (Δ486 mutant). Click here for file

Additional file 6: Table S5 Upstream regulators of KLF11 and mutants. Click here for file

Additional file 7: Figure S2 qPCR validation of Affymetrix gene expression data. A small subset of significantly regulated genes identified by Affymetrix whole-genome microarray for wild type KLF11 or the A347S, Δ486, and EAPP mutants were validated by qPCR. Click here for file
