# Supplementary figures and images for: Single and combinatorial chromatin coupling events underlies the function of transcript factor krüppel-like factor 11 in the regulation of gene networks
Source: BMC Mol Biol. 2014 May 25;15:10. doi: 10.1186/1471-2199-15-10 (PMC4049485; doi:10.1186/1471-2199-15-10)

**EV      WT      A347S       $\Delta$ 486      EAPP**

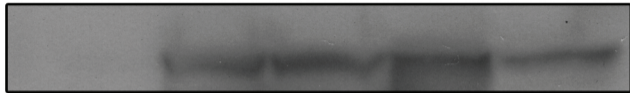

**HIS-Tag**

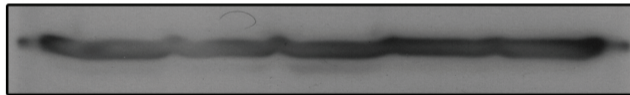

**$\alpha$ -Tubulin**

Supplement: Additional file 1: Figure S1 — Analysis of wild type KLF11 and mutant protein expression. Protein expression of epitope-tagged (His6-Xpress™) wild type KLF11 and the A347S, Δ486, and EAPP mutants demonstrating similar overexpression in Panc1 cells. α-Tubulin was used as a loading control. [file 1471-2199-15-10-S1.pdf]

## KLF11

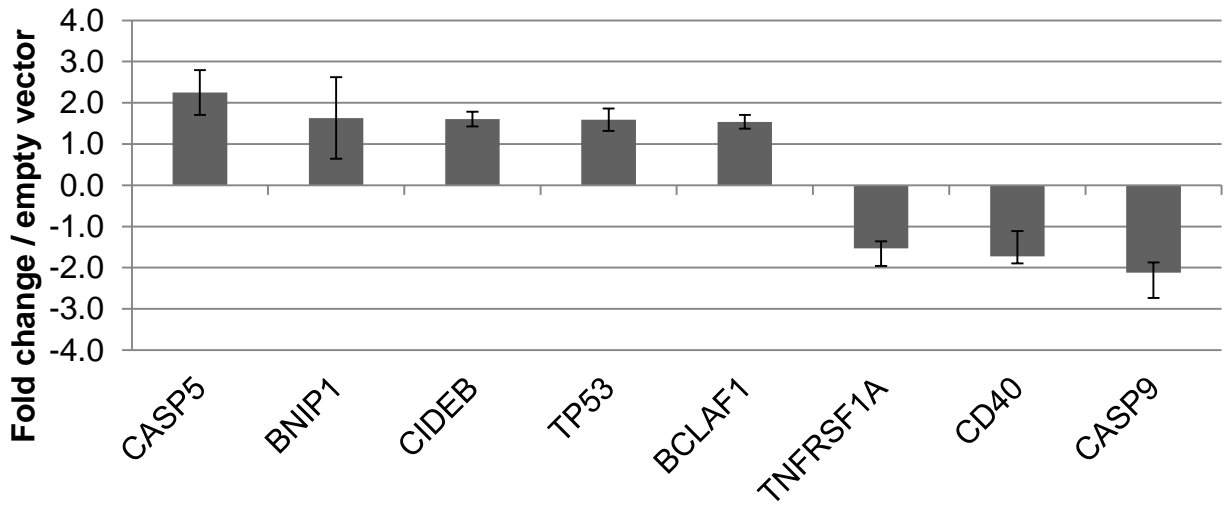

## A347S

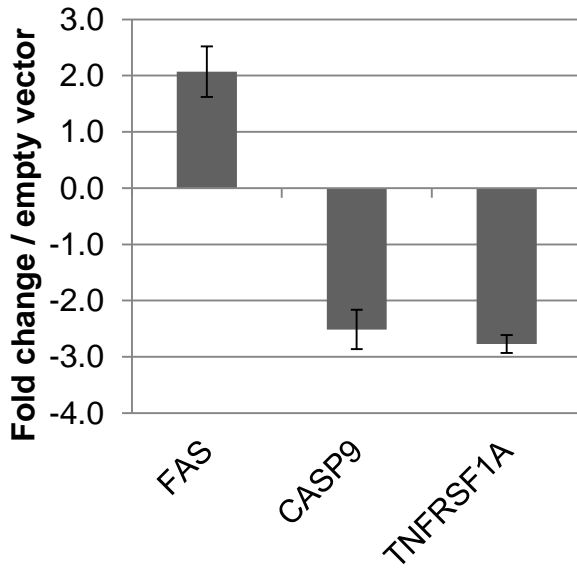

## Δ486

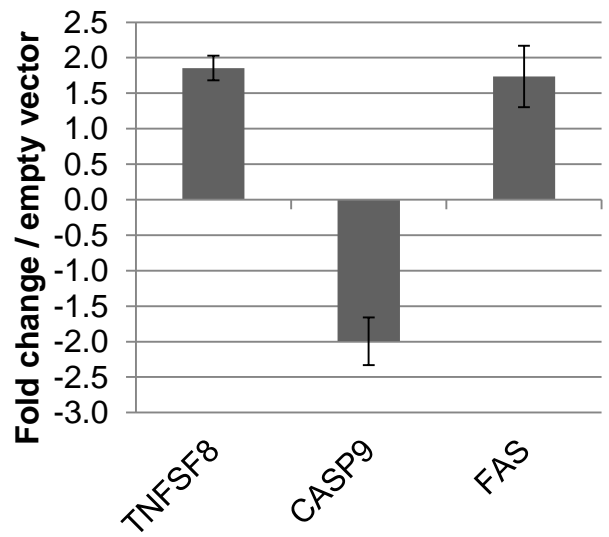

## EAPP

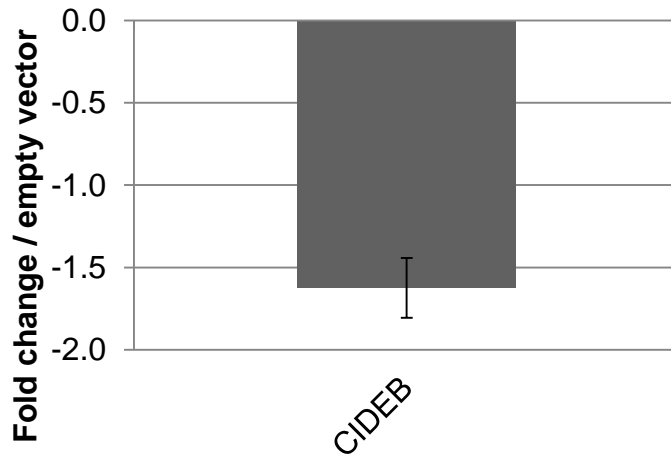

Supplement: Additional file 7: Figure S2 — qPCR validation of Affymetrix gene expression data. A small subset of significantly regulated genes identified by Affymetrix whole-genome microarray for wild type KLF11 or the A347S, Δ486, and EAPP mutants were validated by qPCR. [file 1471-2199-15-10-S7.pdf]
