# Supplementary material for: Single and combinatorial chromatin coupling events underlies the function of transcript factor krüppel-like factor 11 in the regulation of gene networks
Source: BMC Mol Biol. 2014 May 25;15:10. doi: 10.1186/1471-2199-15-10 (PMC4049485; doi:10.1186/1471-2199-15-10)
Supplement: Additional file 2: Table S1 — Overlapping biological function mediated by KLF11 and mutants. [file 1471-2199-15-10-S2.docx]

**Supplemental Table 1: Overlapping biological function mediated by KLF11 and mutants.**

|  |  | **Number of genes** | | | | **Enrichment Score** | | | |
| --- | --- | --- | --- | --- | --- | --- | --- | --- | --- |
| **GO ID** | **Common to KLF11, A347S, Δ486, and EAPP** | **KLF11** | **Δ486** | **A347S** | **EAPP** | **KLF11** | **Δ486** | **A347S** | **EAPP** |
| 44255 | cellular lipid metabolic process | 15 | 16 | 10 | 2 | 15.24 | 9.15 | 9.61 | 4.01 |
|  |  |  |  |  |  |  |  |  |  |
|  |  | **Number of genes** | | | | **Enrichment Score** | | | |
| **GO ID** | **Common to KLF11 and A347S and Δ486 mutants** | **KLF11** | **Δ486** | **A347S** | **EAPP** | **KLF11** | **Δ486** | **A347S** | **EAPP** |
| 6103 | 2-oxoglutarate metabolic process | 5 | 4 | 3 | 1 | 9.81 | 4.89 | 5.56 | 3.40 |
| 19432 | triglyceride biosynthetic process | 6 | 7 | 4 | 1 | 9.30 | 7.84 | 6.09 | 3.50 |
| 8652 | cellular amino acid biosynthetic process | 5 | 5 | 3 | 1 | 6.92 | 4.29 | 3.93 | 3.27 |
| 16139 | glycoside catabolic process | 2 | 2 | 2 | 1 | 6.47 | 5.25 | 7.07 | 3.95 |
| 46477 | glycosylceramide catabolic process | 2 | 2 | 2 | 1 | 6.47 | 5.25 | 7.07 | 3.73 |
| 43497 | regulation of protein heterodimerization activity | 2 | 2 | 2 | 1 | 4.91 | 3.72 | 5.49 | 3.50 |
| 35338 | long-chain fatty-acyl-CoA biosynthetic process | 3 | 4 | 2 | 1 | 4.89 | 5.09 | 3.30 | 4.48 |
| 55114 | oxidation-reduction process | 11 | 18 | 9 | 1 | 4.55 | 5.38 | 4.50 | 3.31 |
| 43496 | regulation of protein homodimerization activity | 2 | 2 | 2 | 1 | 4.32 | 3.16 | 4.89 | 3.50 |
| 51607 | defense response to virus | 4 | 5 | 3 | 1 | 4.10 | 3.36 | 3.31 | 3.31 |
| 16192 | vesicle-mediated transport | 9 | 18 | 8 | 1 | 3.58 | 6.92 | 4.24 | 3.95 |
| 6081 | cellular aldehyde metabolic process | 2 | 3 | 2 | 1 | 3.52 | 4.37 | 4.08 | 4.64 |
| 61098 | positive regulation of protein tyrosine kinase activity | 2 | 3 | 3 | 1 | 3.52 | 4.37 | 6.95 | 3.80 |
| 43627 | response to estrogen stimulus | 4 | 8 | 6 | 1 | 3.03 | 5.60 | 7.54 | 3.50 |
