# Supplementary material for: Single and combinatorial chromatin coupling events underlies the function of transcript factor krüppel-like factor 11 in the regulation of gene networks
Source: BMC Mol Biol. 2014 May 25;15:10. doi: 10.1186/1471-2199-15-10 (PMC4049485; doi:10.1186/1471-2199-15-10)
Supplement: Additional file 3: Table S2 — Top biological processes mediated by KLF11 and mutants compared to empty vector. [file 1471-2199-15-10-S3.docx]

**Supplemental Table 2: Top biological processes mediated by KLF11 and mutants compared to empty vector.**

| **KLF11** | **EAPP** | **Δ486** | **A347S** |
| --- | --- | --- | --- |
| cellular lipid metabolic process | nitric oxide transport | cellular lipid metabolic process | cellular lipid metabolic process |
| 2-oxoglutarate metabolic process | olfactory bulb interneuron differentiation | positive regulation of protein binding | G2/M transition DNA damage checkpoint |
| triglyceride biosynthetic process | thymine catabolic process | triglyceride biosynthetic process | response to estrogen stimulus |
| response to insulin stimulus | negative regulation of glycolysis | proton transport | glycoside catabolic process |
| cellular amino acid biosynthetic process | steroid catabolic process | response to hypoxia | glycosylceramide catabolic process |
| glycoside catabolic process | ketone body catabolic process | vesicle-mediated transport | positive regulation of protein tyrosine kinase activity |
| glycosylceramide catabolic process | outer ear morphogenesis | positive regulation of cell growth | triglyceride biosynthetic process |
| tricarboxylic acid cycle | UDP-N-acetylglucosamine biosynthetic process | cellular nitrogen compound metabolic process | response to DNA damage stimulus |
| glucose metabolic process | cellular ketone body metabolic process | erythrocyte development | 2-oxoglutarate metabolic process |
| negative regulation of telomere maintenance | ketone body biosynthetic process | ER to Golgi vesicle-mediated transport | regulation of protein heterodimerization activity |
| fatty acid beta-oxidation | response to nutrient | tricarboxylic acid cycle | response to cholesterol |
| response to heat | response to ethanol | embryonic digit morphogenesis | response to ionizing radiation |
| negative regulation of IGFR signaling pathway | glycerol-3-phosphate metabolic process | response to estrogen stimulus | positive regulation of protein binding |
| cellular response to stimulus | mesenchymal to epithelial transition | polyamine metabolic process | positive regulation of DNA repair |
| biosynthetic process | valine metabolic process | oxidation-reduction process | proton transport |
| positive regulation of cell growth | regulation of neural precursor cell proliferation | chromatin remodeling | induction of apoptosis by intracellular signals |
| cellular nitrogen compound metabolic process | forelimb morphogenesis | post-embryonic development | regulation of transcription, DNA-dependent |
| isocitrate metabolic process | hindlimb morphogenesis | glycoside catabolic process | regulation of protein homodimerization activity |
| regulation of protein heterodimerization activity | fructose 6-phosphate metabolic process | glycosylceramide catabolic process | response to folic acid |
| L-methionine salvage from methylthioadenosine | oxygen transport | myeloid leukocyte differentiation | oxidation-reduction process |
