# Supplementary material for: Single and combinatorial chromatin coupling events underlies the function of transcript factor krüppel-like factor 11 in the regulation of gene networks
Source: BMC Mol Biol. 2014 May 25;15:10. doi: 10.1186/1471-2199-15-10 (PMC4049485; doi:10.1186/1471-2199-15-10)
Supplement: Additional file 4: Table S3 — Biological processes unique to the decoupling of KLF11 from WD40 proteins (A347S mutant). [file 1471-2199-15-10-S4.docx]

**Supplemental Table 3: Biological processes unique to the decoupling of KLF11 from WD40 proteins (A347S mutant)**

| **List 1** | **List 2** | **List 3** |
| --- | --- | --- |
| chromatin modification  chromatin silencing at rDNA  DNA replication  DNA-dependent DNA replication initiation  negative regulation of centrosome duplication  nucleotide-excision repair  positive regulation of DNA repair  telomere maintenance  telomere maintenance via telomere lengthening  telomere maintenance via telomere shortening  protection from non-homologous end joining at telomere  protein localization to chromosome, telomeric region | negative regulation of protein autophosphorylation  protein dephosphorylation  histone H3 deacetylation  methylation  histone H3-K4 demethylation | transcription elongation from RNA polymerase II promoter  transcription initiation from RNA polymerase II promoter regulation of transcription from RNA polymerase II promoter in response to oxidative stress  positive regulation of transcription from RNA polymerase I promoter  regulation of transcription from RNA polymerase I promoter  termination of RNA polymerase I transcription  mRNA transport |
