# Supplementary material for: Single and combinatorial chromatin coupling events underlies the function of transcript factor krüppel-like factor 11 in the regulation of gene networks
Source: BMC Mol Biol. 2014 May 25;15:10. doi: 10.1186/1471-2199-15-10 (PMC4049485; doi:10.1186/1471-2199-15-10)
Supplement: Additional file 5: Table S4 — Biological processes unique to the decoupling of KLF11 from HP1a/HMT (Δ486 mutant). [file 1471-2199-15-10-S5.docx]

**Supplemental Table 4: Biological processes unique to the decoupling of KLF11 from HP1a/HMT (Δ486 mutant).**

| **List 1** | **List 2** |
| --- | --- |
| beta-amyloid metabolic process  positive regulation of triglyceride biosynthetic process  L-serine metabolic process  porphyrin metabolic process  heme biosynthetic process  sterol biosynthetic process  polyamine biosynthetic process  dopamine biosynthetic process  folic acid metabolic process  response to cholesterol  response to glucose stimulus  aromatic amino acid family metabolic process response to iron ion  electron transport chain  regulation of proteasomal protein catabolic process  protein deubiquitination  protein modification process  positive regulation of protein export from nucleus  protein import into nucleus, translocation  positive regulation of protein import into nucleus, translocation  protein autophosphorylation  vesicle docking involved in exocytosis  actin cytoskeleton reorganization  protein tetramerization | positive regulation of epidermal growth factor receptor signaling pathway  positive regulation of epithelial to mesenchymal transition  inner cell mass cell proliferation  positive regulation of epithelial cell migration  cellular response to estradiol stimulus  negative regulation of survival gene product expression  negative regulation of growth  induction of apoptosis by intracellular signals  cell death |
