# Supplementary material for: Single and combinatorial chromatin coupling events underlies the function of transcript factor krüppel-like factor 11 in the regulation of gene networks
Source: BMC Mol Biol. 2014 May 25;15:10. doi: 10.1186/1471-2199-15-10 (PMC4049485; doi:10.1186/1471-2199-15-10)
Supplement: Additional file 6: Table S5 — Upstream regulators of KLF11 and mutants. [file 1471-2199-15-10-S6.docx]

**Supplemental Table 5: Upstream regulators of KLF11 and mutants.**

| **KLF11 URs** | **State** | **Z-score** | **p-value** | **Target molecules in dataset** |
| --- | --- | --- | --- | --- |
| PPARG | Inhibited | -2,323 | 9,74E-03 | BACE1,BDH1,CPT1A,ERO1L,FASN,GPAM,HYOU1,IRAK4,IVD,KRT19,PC,PCTP,SLC25A1,SLC2A1,UCK1 |
| HNF1A | Inhibited | -2,000 | 8,90E-02 | ACAT2,ATG2B,CBS,CCBL2,FBXO8,GATM,GLA,GOT1,GPR39,KIF20A,KLF11,MON1B,SFXN2,SUPV3L1 |

| **A347S URs** | **State** | **Z-score** | **p-value** | **Target molecules in dataset** | **Mechanistic Network** |
| --- | --- | --- | --- | --- | --- |
| PPARG | Inhibited | -3,585 | 2,82E-04 | ACAA1,ACACA,ANGPTL4,ATP6V1D,BACE1,BCL6,BDH1,CA2,CAT,CCPG1,CPT1A,ERO1L,F11R,FASN,GPAM,HES1,HYOU1,IL12A,INSIG1,IRAK4,IVD,JUN,KLF6,KRT19,LNPEP,NDUFA5,ODC1,PC,PCTP,PDHB,PMM1,SAT1,SCD,SDC1,SLC25A1,SLC25A20,SLC2A1,SORBS1,SREBF1,TGFBR1,TJP1,TKT,UCK1,UCP2,VEGFA |  |
| RXRA | Inhibited | -3,386 | 1,55E-01 | ACACA,ACSL3,ARL4C,CAT,CLMN,CPT1A,FADS2,FASN,HIF1A,HSD17B4,ILK,INSIG1,MID1IP1,PC,RARG,SAT1,SCD,SDC1,SORBS1,SREBF1,TGFB2,VEGFA |  |
| STAT4 | Inhibited | -3,323 | 1,69E-02 | ACSS1,ADSS,ALDOC,ARFGAP3,ATF4,ATP7A,EPOR,ERO1L,ERRFI1,FZD7,GRTP1,HILPDA,HSPA1A/HSPA1B,ING2,LPIN1,LRRFIP1,MBOAT2,P4HA2,PRDX6,RNF128,SAT1,SERPINB1,SETD5,STAT1,VEGFA | FOXO4,HIF1A,TP53 |
| HIF1A | Inhibited | -3,257 | 3,56E-03 | ALDOC,ANGPTL4,BACE1,BNIP3,BNIP3L,CD24,CHKA,CYB5A,EMC9,EPAS1,EPOR,ERGIC1,ERO1L,HIF1A,HILPDA,HIST1H4A (includes others),HIST2H2AC,ITPR1,JUN,JUP,KDM3A,KRT19,LIFR,MCL1,P4HA1,P4HA2,PFKL,QKI,SDC4,SIRT2,SLC25A37,SLC29A1,SLC2A1,TAF9B,TGFB2,VEGFA | HIF1A,PPARG,SREBF1,SREBF2,TP53 |
| PPARGC1A | Inhibited | -2,930 | 1,00E00 | ACACA,CALM1 (includes others),CAT,CPT1A,FASN,LPIN1,SCD,SLC25A20,SREBF1,UCP2 | PPARG,SCAP,SREBF1,SREBF2,TP53 |
| SREBF2 | Inhibited | -2,875 | 2,18E-03 | ACACA,ALDOC,CYB5A,DHCR7,FADS2,FASN,IDH1,INSIG1,LSS,SCD,SREBF1,STARD4,TM7SF2 | HIF1A,PPARG,SREBF1,SREBF2,TP53 |
| ERG | Inhibited | -2,828 | 3,34E-01 | ADD1,ARHGAP17,CDC42BPB,DBN1,ILK,PIM1,PTPN4,RAB2A,RASA2 | FOXO4,HIF1A,HIF3A,TP53 |
| ATF4 | Inhibited | -2,736 | 1,74E-01 | ATF4,CPOX,DDIT4,ERO1L,JUN,LGALS3,MCL1,MID1IP1,PYCR1,SLC7A1,VEGFA |  |
| JUN | Inhibited | -2,660 | 1,00E00 | ACAT2,CCND2,CYP1B1,DKK1,ERCC4,EZR,FAS,GTF2B,HES1,HLA-B,JUN,LGALS3,LMNA,MTHFR,PARD6B,PPP2R2A,RARG,SCD,SDC1,SGK1,SLC6A6,SLC7A1,STAT1,STMN1,VEGFA |  |
| NFE2L2 | Inhibited | -2,587 | 1,00E00 | ABCC4,AKR1A1,ATF4,BNIP3,C5,CAT,CCRN4L,DCTN3,DHCR7,EPAS1,EPB41,ESD,IFNGR2,LMNA,NQO2,SAT1,SEC23A,SLC1A4,SLC2A1,SREBF1,SYT1,TBRG1,TCN2,UGDH,USP14,VEGFA | FOXO4,HIF1A,PTEN,TP53 |
| MITF | Inhibited | -2,540 | 3,49E-01 | ASAH1,CCNG2,CHKA,GM2A,HIF1A,ITGA3,IVNS1ABP,LGALS3,PSEN2,SDC1,SLC19A2,SORT1,TFAP2A | ERBB2,FOXO4,HIF1A,PPARG,PTEN,TP53,TSC2 |
| SREBF1 | Inhibited | -2,530 | 1,79E-03 | ACACA,ALDOC,CYB5A,DHCR7,DPY19L3,ELOVL7,FADS2,FAS,FASN,GPAM,HSPA1A/HSPA1B,IDH1,IL12A,INSIG1,LGALS3,LPIN1,LSS,SCD,SREBF1,STARD4,SUCLG1,TM7SF2,UCP2,VEGFA |  |
| NR1H3 | Inhibited | -2,453 | 1,28E-01 | ACACA,ACSL3,ARL4C,FASN,GPAM,MID1IP1,SCD,SREBF1,VEGFA |  |
| ARNT | Inhibited | -2,425 | 1,63E-01 | BNIP3,CCND2,CYP1B1,ERO1L,HIF1A,KIF20A,MYO1C,SLC2A1,VEGFA |  |
| GLI1 | Inhibited | -2,268 | 5,23E-02 | ASPM,CCND2,CD24,CMBL,DKK1,EZR,IMPA2,INSIG1,JUP,KRT19,LMNA,MRPS6,NQO2,PIM1,PLOD1,PPAP2C,RPS6KA1,VEGFA,ZC3HAV1L |  |
| NR1H2 | Inhibited | -2,236 | 1,54E-01 | ACACA,FASN,HSD17B4,LRP8,SCD,SREBF1,VEGFA |  |
| RORA | Inhibited | -2,236 | 3,59E-01 | ADIPOR1,ARNTL,CCRN4L,CYP2C18,ELOVL7,FASN,GSTM2,HSD17B7,SCD,SLC16A10,SLC2A13,SREBF1,UCP2 |  |
| MLXIPL | Inhibited | -2,195 | 2,52E-02 | ACACA,CPT1A,FASN,MID1IP1,SCD |  |
| SP1 | Inhibited | -2,125 | 1,31E-01 | ACSS1,ALDH3A2,BACE1,BNIP3L,CAT,CBS,CCND2,CD99,CDC25B,CYP1B1,EPOR,EZR,FAS,FASN,HDAC1,HIF1A,HIST1H4A (includes others),HSD17B7,HSPA1A/HSPA1B,IL12A,ITGA2,JUN,KRT19,MAT2B,MCL1,MSH6,OGG1,PIM1,RBL1,RECK,SETDB1,SGK1,SLC2A1,SLC39A8,SLC7A1,SMAD3,SREBF1,STAT1,TGFB2,TGFBR1,UGDH,VEGFA |  |
| PPARA | Inhibited | -2,009 | 2,70E-02 | ACAA1,ACACA,ALDH3A2,ANGPTL4,C1S,C5,CAT,CHKA,CPT1A,CYP2C18,DHCR7,F11R,FADS2,FASN,GPAM,GPD2,HLA-E,HSD17B4,INSIG1,KIF20A,LIFR,LSS,OGG1,PC,PCTP,PPM1D,PRDX6,QPCT,RAD51B,RETSAT,SAT1,SCD,SLC25A20,SLC27A2,SOCS2,SREBF1,TJP1,UCP2,VEGFA |  |
| E2f | Inhibited | -2,000 | 1,13E-01 | EPAS1,HDAC1,HIF1A,HIST1H3A (includes others),HIST1H4A (includes others),ITGA6,MFAP1,MIR17HG,RBBP5,RBL1,RECQL,TRMT13 |  |
| PXR ligand-PXR-Retinoic acid-RXRÎ± | Inhibited | -2,000 | 2,87E-01 | ALDH3A2,CAT,GSTM2,SCD |  |
| PPRC1 | Inhibited | -2,000 | 4,40E-01 | ATF4,DDIT4,ERRFI1,NAMPT |  |
| GFI1 | Activated | 2,646 | 3,38E-01 | ATF1,ETS2,JUN,KAT2B,RIPK1,SMAD3,STAT1,TNFRSF1A |  |
| STAT1 | Activated | 2,538 | 1,00E00 | CCND2,FAS,HIF1A,IFI27,IL12A,JUN,PIM1,SAMHD1,SLFN5,SMAD2,SMAD3,STAT1,USP18 |  |
| PIAS1 | Activated | 2,449 | 1,40E-02 | ACACA,FASN,MCL1,SCD,SREBF1,STAT1 |  |
| HOXD10 | Activated | 2,449 | 3,43E-01 | EZR,HBEGF,ITGA3,NCS1,TJP1,USP14 |  |
| SPDEF | Activated | 2,449 | 3,89E-01 | HIF1A,ITGA3,ITGA6,SDC1,SMAD2,SMAD3 |  |
| HIC1 | Activated | 2,345 | 2,14E-02 | AHNAK2,CA2,CCDC176,ITPR1,LRP8,PLEC,SIRT1,SNAPC1,TNS3,WDR6 |  |
| EZH2 | Activated | 2,141 | 1,00E00 | ANXA6,CCND2,CLDN10,CYB5R2,CYP1B1,DDT,DKK1,EZR,FUCA1,SIRT1,TBX3,WTAP |  |
| VHL | Activated | 2,137 | 1,00E00 | ATF4,BNIP3,CLDN4,EPAS1,HIF1A,LMNA,SLC2A1,VEGFA |  |
| N-cor | Activated | 2,000 | 1,85E-01 | ACACA,FASN,SCD,SREBF1,USP18 |  |

| **Δ486 URs** | **State** | **Z-score** | **p-value** | **Target molecules in dataset** | **Mechanistic Network** |
| --- | --- | --- | --- | --- | --- |
| RXRA | Inhibited | -3,104 | 4,65E-02 | ACACA,ARL4C,CAT,CLMN,CPT1A,FADS2,FASN,HIF1A,HSD17B4,MID1IP1,SAT1,SCD,SORBS1,SREBF1,TGFB2 | FOXO3,FOXO4,Insulin,PPARG,SREBF1,SREBF2,TP53,arachidonic acid |
| PPARGC1A | Inhibited | -2,774 | 1,34E-01 | ACACA,CALM1 (includes others),CAT,CPT1A,FASN,LPIN1,SCD,SREBF1,UCP2 |  |
| PPARG | Inhibited | -2,652 | 7,65E-05 | ACACA,BACE1,BCL6,BDH1,CA2,CAT,CCPG1,CPT1A,ERO1L,FASN,GPAM,HYOU1,IL12A,IRAK4,IVD,KRT19,LNPEP,ODC1,PCTP,PDHB,PMM1,SAT1,SCD,SLC25A1,SLC2A1,SORBS1,SREBF1,TGFBR1,UCK1,UCP2 | FOXO3,PPARG,SREBF1,SREBF2,TP53 |
| NFE2L2 | Inhibited | -2,578 | 1,64E-01 | ABCC4,AKR1A1,BNIP3,C5,CAT,DCTN3,DHCR7,EPB41,ESD,IFNGR2,NQO2,SAT1,SEC23A,SLC1A4,SLC2A1,SREBF1,UGDH,USP14 |  |
| GLI1 | Inhibited | -2,574 | 5,22E-01 | CD24,IMPA2,KRT19,NQO2,PIM1,PLOD1,ZC3HAV1L |  |
| MITF | Inhibited | -2,414 | 1,00E00 | GM2A,HIF1A,IVNS1ABP,LGALS3,PSEN2,SORT1 |  |
| ATF4 | Inhibited | -2,200 | 1,33E-01 | CPOX,DDIT4,ERO1L,LGALS3,MID1IP1,PYCR1,SLC7A1 |  |
| MLXIPL | Inhibited | -2,195 | 1,80E-03 | ACACA,CPT1A,FASN,MID1IP1,SCD |  |
| TP73 | Inhibited | -2,138 | 5,02E-01 | ARNTL,CCNG1,CLMN,DLG1,FASN,IDH2,SAT1,UBE2D1 |  |
| SREBF2 | Inhibited | -2,128 | 2,01E-03 | ACACA,CYB5A,DHCR7,FADS2,FASN,IDH1,LSS,SCD,SREBF1 |  |
| NR1H3 | Inhibited | -2,105 | 3,55E-02 | ACACA,ARL4C,FASN,GPAM,MID1IP1,SCD,SREBF1 |  |
| HIF1A | Inhibited | -2,068 | 3,30E-02 | BACE1,BNIP3,BNIP3L,CD24,CYB5A,EMC9,ERGIC1,ERO1L,HIF1A,HILPDA,HIST1H4A (includes others),ITPR1,KRT19,LIFR,PFKL,SIRT2,SLC2A1,TAF9B,TGFB2 |  |
| NR1H2 | Inhibited | -2,000 | 3,00E-02 | ACACA,FASN,HSD17B4,LRP8,SCD,SREBF1 |  |
| ERG | Inhibited | -2,000 | 3,68E-01 | ADD1,CDC42BPB,PIM1,PTPN4,RAB2A |  |
| RORA | Inhibited | -2,000 | 1,00E00 | ARNTL,FASN,SCD,SLC2A13,SREBF1,UCP2 |  |
| HIC1 | Activated | 2,433 | 4,37E-02 | CA2,CCDC176,ITPR1,LRP8,TNS3,WDR6 |  |
| HOXA10 | Activated | 2,236 | 1,00E00 | ATF6B,IDH2,KLF10,SAT1,SCD |  |
| STAT1 | Activated | 2,219 | 1,00E00 | HIF1A,IFI27,IL12A,PIM1,SAMHD1,SLFN5,SMAD2 |  |
| PIAS1 | Activated | 2,000 | 2,22E-02 | ACACA,FASN,SCD,SREBF1 |  |
| N-cor | Activated | 2,000 | 7,71E-02 | ACACA,FASN,SCD,SREBF1 |  |
| GFI1 | Activated | 2,000 | 2,73E-01 | ATF1,ETS2,KAT2B,RIPK1,TNFRSF1A |  |

| **EAPP URs** | **p-value** | **Target molecules in dataset** |
| --- | --- | --- |
| ZNF496 | 1,53E-03 | HBB |
| FOXN2 | 1,53E-03 | HBB |
| HLTF | 3,05E-03 | HBB |
| DLX4 | 4,57E-03 | HBB |
| TIAL1 | 4,57E-03 | HBB |
| KLF7 | 6,09E-03 | HBB |
| ATF4 | 9,55E-03 | DDIT4,STC2 |
| KLF3 | 1,06E-02 | HBB |
| HNRNPD | 1,22E-02 | HBB |
| MAFF | 1,37E-02 | HBB |
| BRCA1 | 1,48E-02 | DDIT4,HBB |
| CEBPG | 1,52E-02 | HBB |
| CBX2 | 1,67E-02 | HBB |
| MTA2 | 1,67E-02 | HBB |
| KLF13 | 1,82E-02 | HBB |
| NSD1 | 1,97E-02 | HBB |
| CTBP2 | 2,27E-02 | HBB |
| ZFPM1 | 2,27E-02 | HBB |
| GLI1 | 2,32E-02 | GREM2,SALL1 |
| COMMD3-BMI1 | 2,57E-02 | HBB |
| MAFK | 2,57E-02 | HBB |
| SIX1 | 2,71E-02 | SALL1 |
| NFE2 | 2,71E-02 | HBB |
| BACH1 | 2,71E-02 | HBB |
| CARM1 | 3,16E-02 | STC2 |
| DRAP1 | 3,60E-02 | HIST1H2BH/HIST1H2BO |
| KLF1 | 4,04E-02 | HBB |
| GTF2B | 4,04E-02 | HIST1H2BH/HIST1H2BO |
| YBX1 | 4,19E-02 | HBB |
| KAT2B | 4,34E-02 | HBB |
| HOXC8 | 4,34E-02 | GREM2 |
